# Identification of benzoquinones in pretreated lignocellulosic feedstocks and inhibitory effects on yeast

**DOI:** 10.1186/s13568-015-0149-9

**Published:** 2015-09-17

**Authors:** Stefan Stagge, Adnan Cavka, Leif J. Jönsson

**Affiliations:** Department of Chemistry, Umeå University, 901 87 Umeå, Sweden

**Keywords:** *Saccharomyces cerevisiae*, Lignocellulosic hydrolysate, Fermentation inhibitor, *p*-Benzoquinone, 2,6-Dimethoxybenzoquinone, 2,4-Dinitrophenylhydrazine (DNPH), Mass spectrometry

## Abstract

Pretreatment of lignocellulosic biomass under acidic conditions gives rise to by-products that inhibit fermenting microorganisms. An analytical procedure for identification of *p*-benzoquinone (BQ) and 2,6-dimethoxybenzoquinone (DMBQ) in pretreated biomass was developed, and the inhibitory effects of BQ and DMBQ on the yeast *Saccharomyces cerevisiae* were assessed. The benzoquinones were analyzed using ultra-high performance liquid chromatography-electrospray ionization-triple quadrupole-mass spectrometry after derivatization with 2,4-dinitrophenylhydrazine. Pretreatment liquids examined with regard to the presence of BQ and DMBQ originated from six different lignocellulosic feedstocks covering agricultural residues, hardwood, and softwood, and were produced through impregnation with sulfuric acid or sulfur dioxide at varying pretreatment temperature (165–204 °C) and residence time (6–20 min). BQ was detected in all six pretreatment liquids in concentrations ranging up to 6 mg/l, while DMBQ was detected in four pretreatment liquids in concentrations ranging up to 0.5 mg/l. The result indicates that benzoquinones are ubiquitous as by-products of acid pretreatment of lignocellulose, regardless of feedstock and pretreatment conditions. Fermentation experiments with BQ and DMBQ covered the concentration ranges 2 mg/l to 1 g/l and 20 mg/l to 1 g/l, respectively. Even the lowest BQ concentration tested (2 mg/l) was strongly inhibitory to yeast, while 20 mg/l DMBQ gave a slight negative effect on ethanol formation. This work shows that benzoquinones should be regarded as potent and widespread inhibitors in lignocellulosic hydrolysates, and that they warrant attention besides more well-studied inhibitory substances, such as aliphatic carboxylic acids, phenols, and furan aldehydes.

## Introduction

Biochemical conversion of lignocellulosic feedstocks typically proceeds through thermochemical pretreatment, enzymatic saccharification of cellulose, and microbial fermentation of sugars to desirable products, such as bioalcohols, bioacids, and other commodities (Viikari et al. 2012; Stephen et al. 2012). The pretreatment is needed to make the feedstock more susceptible to cellulolytic enzymes and to achieve high product yields. Pretreatment with acid catalysts, such as sulfuric acid and sulfur dioxide, is a well-developed technology that is suitable also for more recalcitrant feedstocks (Galbe and Zacchi 2007; Hu and Ragauskas 2012).

Acid pretreatment mainly targets the hemicellulose, which is hydrolyzed to sugars (Galbe and Zacchi 2007; Hu and Ragauskas 2012). However, by-products are also formed, and in sufficiently high concentrations they will inhibit fermenting microorganisms, such as yeast and bacteria (Jönsson et al. 2013). Due to product recovery, it is desirable to have a highly concentrated system (Jørgensen et al. 2007), which, however, also will contain high concentrations of inhibitors.

The most studied inhibitors are aliphatic carboxylic acids, phenols, and furan aldehydes (Jönsson et al. 2013). Aliphatic carboxylic acids, such as acetic acids, formic acid, and levulinic acid, are carbohydrate degradation products. Phenolic compounds are formed primarily through partial degradation of lignin, which is a polymer consisting of phenylpropane units. As some extractives are phenolic, they are another source of phenolic compounds. Furan aldehydes, such as furfural and 5-hydroxymethylfurfural (HMF) are dehydration products of sugars.

Model experiments have shown that *p*-benzoquinone (BQ) is strongly inhibitory to yeast (Larsson et al. 2000), but, to our knowledge, benzoquinones have never been reported in pretreated lignocellulose, which is probably due to the lack of adequate analytical procedures. 2,6-Dimethoxyhydroquinone has been reported in a hemicellulose hydrolysate of steam-treated birch (Buchert et al. 1990). Hydroquinone (i.e. benzene-1,4-diol) has been found in an acid hydrolysate of Norway spruce (Larsson et al. 1999), and in acid hydrolysates of grasses, agricultural residues, hardwood, softwood, and agave (Mitchell et al. 2014). Hydroquinone is, however, much less toxic than BQ (Larsson et al. 2000). Larsson et al. (2000) did not find any toxic effect of 1 g/l of hydroquinone, the highest concentration tested, in fermentations with the yeast *Saccharomyces cerevisiae*. The lowest concentration of BQ studied by Larsson et al. (2000) was 20 mg/l, which was sufficiently high to completely inhibit the fermentation process. Thus, both the potential presence of BQ in lignocellulosic hydrolysates and the lowest inhibitory concentration remain to be elucidated. There may also be other benzoquinones in lignocellulose hydrolysates, for instance 2,6-dimethoxybenzoquinone (DMBQ), which could tentatively form through degradation of syringyl (4-hydroxy-3,5-dimethoxyphenyl) units in lignin. Furthermore, DMBQ is chemically closely related to 2,6-dimethoxyhydroquinone. Cavka and Jönsson (2013) studied detoxification reactions with BQ and DMBQ, but did not investigate their presence in lignocellulosic hydrolysates or their toxic effects on yeast. The aims of this investigation were to develop a procedure for identification of simple benzoquinones potentially present in lignocellulosic hydrolysates and to determine the practical relevance through determination of concentrations required to obtain an inhibitory effect on yeast.

## Materials and methods

### Pretreatment of lignocellulosic raw materials

The pretreatment liquids (PLs) used in this study were produced from corn cobs (PL1), sugarcane bagasse (PL2), raw cassava stems (*Manihot esculenta*) (PL3), chipped wood of Norway spruce (*Picea abies*) (PL4), European white birch (*Betula pubescens*) (PL5), and hybrid aspen (*Populus tremula* × *P. tremuloides*) (PL6). The pretreatment of corn cobs, bagasse, spruce and birch was performed by SEKAB E-Technology using a 30-l pretreatment reactor in the Biorefinery Demo Plant in Örnsköldsvik, Sweden. Corn cobs were treated in a continuous mode with addition of 0.54 % (w/w) of H_2_SO_4_, at a temperature of 186 °C (corresponding to 11 bar over-pressure) and with a residence time of 6.4 min. The bagasse was pretreated at a temperature of 188 °C (14 bar over-pressure), after being impregnated with SO_2_ (0.3 kg SO_2_/h), corresponding to around 0.6 % SO_2_/kg of sugarcane bagasse (DW, dry weight). The residence time in the reactor was 10 min, and the resulting pH was 2.1. Unbarked spruce wood chips were treated a temperature of 204 °C, (18 bar over-pressure) with an addition of 1.2–1.3 kg SO_2_/h, corresponding to 1 % SO_2_/kg of spruce wood chips (DW). The residence time in the reactor was 7–8 min, and the resulting pH was 1.4–1.5. Birch wood was pretreated at 190 °C (16 bar over-pressure) with a SO_2_ addition of 0.7 kg/h, corresponding to approximately 0.5 % SO_2_/kg birch wood chips (DW). The residence time was 7 min and the resulting pH was 1.8. Milled aspen and raw cassava stem samples were pretreated in laboratory scale reactors, aspen in a microwave reactor (Biotage Initiator 2.0, Biotage, Uppsala, Sweden) and cassava stems in a stainless steel reactor equipped with an internal thermocouple and a PID module for controlling the temperature. The aspen pretreatment in the microwave system was performed at 165 °C for 10 min with an addition of H_2_SO_4_ that resulted in a final concentration of 1 % (w/w) of the total weight of the reaction mixture. Raw cassava stems were pretreated at 170 °C for 20 min in a 1 % (w/w) aqueous solution of H_2_SO_4_ that was added to the biomass at a liquid-to-solids ratio of 9:1. After pretreatments, all of the resulting pretreated materials were cooled and stored at 4 °C until further use.

### Fermentation

Fermentation experiments were conducted to assess the toxic effects of BQ and DMBQ on common baker’s yeast *Saccharomyces cerevisiae* (Jästbolaget AB, Rotebro, Sweden). The fermentations were performed in 25 ml glass flasks equipped with magnets for stirring and sealed with rubber plugs pierced with cannulas for release of carbon dioxide. The flasks were filled with 23.75 ml of citric buffer solution (0.05 M, pH 5.5) containing 2 % glucose with additions of BQ in the concentration ranges 20 mg/l to 1 g/l (Series A) and 2–20 mg/l (Series B), and additions of DMBQ in the concentration range 20 mg/l to 1 g/l. Reference fermentations with the glucose-based medium but no benzoquinones were included for comparison. Each flask was supplemented with 0.5 ml of a nutrient solution (150 g/l yeast extract, 75 g/l (NH_4_)_2_HPO_4_, 3.75 g/l MgSO_4_·7 H_2_O, 238.2 g/l NaH_2_PO_4_·H_2_O), and 0.75 ml of yeast inoculum. The yeast inoculum was prepared in 750-ml cotton-plugged shake flasks with 300 ml YPD medium (2 % yeast extract, 1 % peptone, 2 % d-glucose). The flasks were inoculated and incubated with agitation at 30 °C for approximately 12 h. The cells were harvested in the late exponential growth phase by centrifugation (Allegra X-22R, Beckman Coulter, Brea, CA, USA) at 1500*g* for 5 min. The cells were resuspended in an appropriate amount of sterile water to achieve an inoculum consisting of 0.2 g/l (cell dry weight) in all fermentation vessels. The flasks were incubated at 30 °C in a water bath with magnetic stirring (IKA-Werke, Staufen, Germany). Samples for measurement of sugars and ethanol were withdrawn during the fermentation. The glucose levels during fermentation experiments were estimated by using a glucometer (Accu-Chek Active, Roche Diagnostics, Basel, Schwitzerland). Glucometer data were corrected using glucose values obtained with an Agilent 1200 series high-performance liquid chromatography (HPLC) system (Agilent Technologies, Waldbronn, Germany) (see section below).

### Analysis of glucose consumption and ethanol formation

The consumption of glucose and the production of ethanol were determined off-line using an Agilent Technologies 1200 series HPLC system. The device was equipped with an autosampler, a refractive index detector (RID), a binary pump, and a degasser, all from the Agilent 1200 series. The chromatographic separation was performed using a Bio-Rad Aminex HPX-87H column (Bio-Rad Laboratories AB, Solna, Sweden). Separation was achieved with an isocratic gradient of 0.01 N H_2_SO_4_ at a flow-rate of 0.6 ml/min and the temperature of the column oven was set to 60 °C. The temperature of the detector cell of the RID was set to 55 °C (Sluiter et al. 2008). An external calibration approach was applied for quantification.

### Sample preparation and derivatization for determination of *p*-benzoquinone (BQ) and 2,6-dimethoxy-1,4-benzoquinone (DMBQ)

DNPH (2,4-dinitrophenylhydrazine) was recrystallized twice with hot ACN (acetonitrile) to remove possible contaminants before the derivatization (EPA 1996). The reagent solution was prepared as described by Kieber and Mopper (1990) except that 36 mg of DNPH were used instead of 20 mg. All samples were centrifuged and, if necessary, diluted with Milli-Q water (Millipore, Billerica, MA, USA) prior to the derivatization with DNPH. All samples and reference standards were handled as described below.

A stock solution for calibration samples was prepared by incubation of 10 µl of BQ (1.39 mM) and 15 µl DMBQ (0.89 mM) together with 1500 µl of the DNPH reagent solution in a capped glass vial at RT overnight. One hundred μl of each of the six PLs were incubated with 1500 µl of the DNPH reagent solution and kept at RT overnight. After 16 h of incubation, each of the derivatized samples (the stock solution for calibration and the six PLs) were diluted with ACN to a final volume of 5 ml.

Before the UHPLC-ESI-QqQ-MS (ultra-high performance liquid chromatography-electrospray ionization-triple quadrupole-mass spectrometry analysis), an equidistant multipoint calibration curve was prepared from the stock solution for calibration samples. The concentration ranges covered by the calibration samples were: BQ, 0.011–1.665 µM; DMBQ, 0.011–0.267 µM. The results of the regression analysis were used to calculate concentrations of each analyte.

### UHPLC-ESI-TripleQuad-MS analysis

All UHPLC-ESI-QqQ-MS analyses were carried out with an Agilent 1290 Infinity instrument consisting of an auto-sampler, a gradient pump, a degasser, and a column oven. The HPLC was coupled to an Agilent 6490 TripleQuad mass spectrometer (QqQ-MS) used in negative electrospray-ionization (ESI) mode and equipped with Agilent Jetstream technology. The mass spectrometer (MS) source parameters were optimized for each compound for the two most abundant multiple reaction monitoring (MRM) transitions under flow injection acquisition (FIA) mode using the following final settings: gas temperature 290 °C, gas flow 20 l/min, nebulizer 30 psi, sheath gas temperature 400 °C, sheath gas flow 12 l/min, capillary voltage −3000 V, nozzle voltage −2000 V. The iFunnel for the high and low pressure RF (reflector) were 150 and 40 V, respectively. Chromatographic separation was achieved with a 2.1 mm × 150 mm XTerra MS C18 5 µm column (Waters, Milford, MA, USA). A gradient profile was developed using aqueous 0.1 % (v/v) formic acid as eluent A, and a mixture of ACN/2-propanol (75/25 % v/v) with 0.1 % formic acid as eluent B. With these eluents, a satisfactory separation could be achieved using the following conditions: 0.0–9.0 min 30–40 % B, 9.0–17.0 min 40–50 % B, 17.0–21.0 min 50–70 % B, 21.0–21.01 min 70–90 % B, 21.01–27.0 min 95 % B, 27.0–27.01 min 95–30 % B, 27.01–31.0 min 30 % B and, at the end, 2 min post-time for further re-equilibration. The flow rate was kept constant at 0.25 ml/min. The column temperature was 30 °C. Five µl of each sample were injected.

The Agilents MassHunter workstation software was used for quantitative analysis of the LC–MS/MS results (Agilent Technologies 2008, Version B.05.02). Two different time windows for the determination of the noise via a root-mean-square algorithm were defined before and after each MRM-transition signal. The presence of a compound in a sample and the confirmation of its identity are based on signal/noise-ratios (S/N) greater than 3 (lower limit of detection, LOD) and the presence of a qualifier transition mass unique for the specific compound.

## Results

The potential presence of BQ and DMBQ in a collection of six lignocellulosic hydrolysates was investigated, and the toxic effects of the two benzoquinones on the yeast *S. cerevisiae* were compared. In order to do this, an analytical approach to detect and quantify BQ and DMBQ in lignocellulosic hydrolysates was first elaborated. The hydrolysates were derived from different types of lignocellulosic feedstocks including agricultural residues, softwood, and hardwood. The pretreatment of the lignocellulosic feedstocks was performed using different acid catalysts (sulfuric acid and sulfur dioxide), temperatures ranging from 165 to 204 °C, and with a residence time ranging from 6 to 20 min (Table 1). The use of this variety of different feedstocks and pretreatment conditions should provide a comprehensive view of the prevalence of benzoquinones in pretreated lignocellulosic biomass.

**Table 1:** Pretreatment liquids and contents of BQ and DMBQ

Pretreatment liquid	Feedstock	Pretreatment catalyst and conditions	[BQ] (mg/l)	[DMBQ] (mg/l)
PL1	Corn cobs	H_2_SO_4_, 186 °C, 6.4 min	2.006 ± 0.231	0.230 ± 0.028
PL2	Sugarcane bagasse	SO_2_, 188 °C, 10 min	6.138 ± 0.838	<LOD
PL3	Cassava stems	H_2_SO_4_, 170 °C, 20 min	5.167 ± 1.454	0.200 ± 0.049
PL4	Norway spruce	SO_2_, 204 °C, 7-8 min	4.156 ± 0.381	<LOD
PL5	Birch	SO_2_, 190 °C, 7 min	<LOQ	0.387 ± 0.065
PL6	Aspen	H_2_SO_4_, 165 °C, 10 min	0.638 ± 0.307	0.511 ± 0.101

The derivatization with DNPH was first tested with reference standards dissolved in water as all PLs are aqueous solutions. Both the reference standards for BQ and DMBQ were successfully derivatized and the expected masses for precursor ions and product ions were confirmed through QqQ-MS. The sample preparation method and the use of the QqQ-MS detector resulted in satisfactory linearity for calibration of BQ with an R^2^ > 0.99. The quantification of DMBQ was rendered difficult by an unexpected decrease of the signal intensities during analysis. Reducing the number of calibration points from nine to four by excluding the highest concentration levels gave an acceptable linearity with R^2^ = 0.96, although resulting in reduced precision in the determination of DMBQ in the PLs. The analytical procedure nevertheless resulted in good sensitivity with the LOD (limit of detection) and the LQQ (limit of quantification) being in the lower nanomolar range for both BQ and DMBQ (see Table 2).

**Table 2:** Results and parameters of the UHPLC-ESI-QqQ-MS analysis

Compound	Rt (min)	Precursor ion	Transition^a^	CE (eV)	Cell Acc (V)	R^2^	Slope	LOD/LOQ (µmol/l)
*p*-Benzoquinone								
	17.5	286.9	166.8	15	3	0.99	28,263	2.939 × 10^−3^
			108.9					9.979 × 10^−3^
2,6-Dimethoxy-1,4-benzoquinone^b^								
	14.4	346.8	166.8	20	3	0.96	347,852	7.116 × 10^−3^
			108.8					2.135 × 10^−3^

*Rt* retention time, *CE* collision energy, *Cell Acc* cell acceleration, *LOD & LOQ* limits of dectection and quantification

^a^The upper (first row) transition masses were used for quantification and the lower (second row) for qualification purposes

^b^The R^2^ and slope values for DMBQ are derived from a four-point calibration

**Fig. 1:**
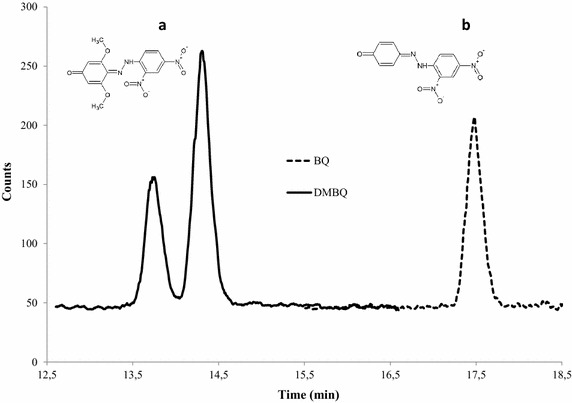
Chromatogram from MRM transitions of DNPH-hydrazones of **a** DMBQ (figure showing one of two possible 2,4-dinitrophenylhydrazone structural isomers that could give rise to the double peak) (m/z 346.8 → 166.8) and **b** BQ (m/z 286.9 → 166.8) in PL6 (aspen)

BQ was detected and quantified in five out of the six PLs studied (PL1-4, PL6) (Table 1; Fig. 1). BQ was detected also in PL5, but, since the S/N ratio was within the range 3–10, the concentration of BQ in PL5 was <LOQ (see Tables 1, 2). The concentration of BQ in PLs 1–4 and PL6 ranged from about 0.6 mg/l (~6 μM) in PL6 (aspen) to about 6 mg/l (~60 μM) in PL2 (sugarcane bagasse) (Table 1). Thus, BQ was found in different kinds of pretreated biomass (agricultural residues, softwood, hardwood), and after pretreatment with different acidic catalysts (SO_2_ and H_2_SO_4_) using different temperatures and residence times. This indicates that BQ is ubiquitous in lignocellulosic biomass after pretreatment under acidic conditions.

DMBQ was detected and quantified in four out of six PLs (PL1, PL3, PL5-6) (Table 1; Fig. 1). With regard to PL2 and PL4, the content of DMBQ was <LOD. The highest amount of DMBQ, ~0.5 mg/l (~3 μM), was found in PL6 (Fig. 1). With the exception of PL5, which contained ~0.4 mg/l DMBQ, the content of BQ in a given PL was always higher than the content of DMBQ.

**Fig. 2:**
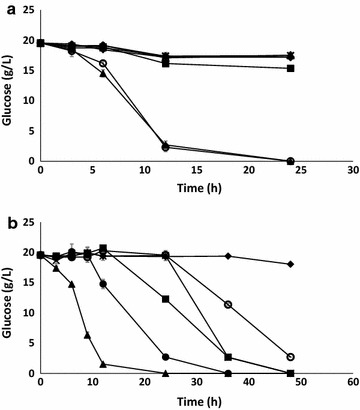
Glucose consumption by *S. cerevisiae* during fermentation of medium without addition of benzoquinones (reference fermentation) (*filled triangle*) and with additions of **a** 0.02 g/l BQ (*filled diamond*), 0.20 g/l BQ (*unfilled diamond*), 1.00 g/l BQ (*unfilled triangle*), 0.02 g/l DMBQ (*unfilled circle*), 0.20 g/l DMBQ (*filled crossmark*), and 1.00 g/l DMBQ (*filled square*), and **b** 2 mg/l BQ (*filled circle*), 5 mg/l BQ (*filled square*), 10 mg/l BQ (*filled crossmark*), 15 mg/l BQ (*unfilled circle*), and 20 mg/l BQ (*filled diamond*). The *gray error bars* indicate the standard deviations of glucose measurements in replicate fermentations

Inhibitory effects on the yeast *S. cerevisiae* were studied in experiments with BQ concentrations ranging from 2 mg/l to 1 g/l, and with DMBQ concentrations ranging from 20 mg/l to 1 g/l (Fig. 2; Table 3). The glucose consumption and the ethanol yield in these experiments were compared with those of reference fermentations with the same glucose-based medium but without any inhibitors.

**Table 3:** Results of fermentation experiments with 2 % glucose and addition of BQ or DMBQ

Compound (g/l)	Glucose start (g/l)	Glucose at time point *t* (g/l)	Glucose consumed at time point *t* (g/l)	Y_EtOH_ on consumed sugar^a^	BEY^b^	Q_EtOH_^c^	Time point *t* (h)
Experimental series A							
*BQ*							
0.02	19.5 ± 1.9	17.2 ± 1.7	2.4 ± 0.2	0.25 ± 0.03	0.03 ± 0.01	0.05 ± 0.01	12
0.20	19.5 ± 1.9	17.4 ± 1.7	2.2 ± 0.2	0.26 ± 0.03	0.03 ± 0.01	0.05 ± 0.01	12
1.00	19.5 ± 1.9	17.1 ± 1.7	2.5 ± 0.2	0.21 ± 0.02	0.03 ± 0.01	0.04 ± 0.01	12
*DMBQ*							
0.02	19.5 ± 1.9	2.3 ± 0.2	17.6 ± 1.7	0.41 ± 0.04	0.36 ± 0.04	0.59 ± 0.06	12
0.20	19.5 ± 1.9	17.4 ± 1.7	2.2 ± 0.2	0.22 ± 0.02	0.02 ± 0.01	0.04 ± 0.01	12
1.00	19.5 ± 1.9	16.2 ± 1.6	3.4 ± 0.3	0.13 ± 0.01	0.02 ± 0.01	0.04 ± 0.01	12
Reference	19.5 ± 1.9	2.7 ± 0.3	17.3 ± 1.7	0.50 ± 0.05	0.43 ± 0.04	0.70 ± 0.07	12
Experimental series B							
*BQ*							
0.002	19.5 ± 1.9	2.7 ± 0.3	16.9 ± 1.7	0.50 ± 0.05	0.43 ± 0.04	0.35 ± 0.04	24
0.005	19.5 ± 1.9	2.7 ± 0.3	16.9 ± 1.7	0.49 ± 0.05	0.42 ± 0.04	0.23 ± 0.02	36
0.010	19.5 ± 1.9	2.6 ± 0.3	16.9 ± 1.7	0.49 ± 0.05	0.42 ± 0.04	0.23 ± 0.02	36
0.015	19.5 ± 1.9	2.7 ± 0.3	16.9 ± 1.7	0.48 ± 0.05	0.41 ± 0.04	0.17 ± 0.02	48
0.020	19.5 ± 1.9	17.5 ± 1.8	2.1 ± 0.2	0.27 ± 0.03	0.03 ± 0.01	0.03 ± 0.00	48
Reference	19.5 ± 1.9	1.5 ± 0.2	18.0 ± 1.8	0.50 ± 0.05	0.46 ± 0.05	0.75 ± 0.08	12

The table shows the values at start and at a specific time point in the fermentation experiments. The relative standard deviation of the method that was used for monosaccharide and ethanol analysis was estimated to ≤10 %

^a^g EtOH/g consumed glucose

^b^Balanced ethanol yield; g EtOH/g glucose prior to fermentation

^c^Volumetric ethanol productivity; g EtOH × l^−1^ × h^−1^

The first set of experiments with BQ (Series A) showed that concentrations that were 20 mg/l or higher strongly inhibited glucose consumption (Fig. 2a), Y_EtOH_ (ethanol yield) on consumed sugar, BEY (Balanced Ethanol Yield), and Q_EtOH_ (ethanol productivity) (Table 3). In the second set of experiments (Series B) (Fig. 2b; Table 3), lower concentrations of BQ (2–20 mg/l) were studied. As Fig. 2b shows, all concentrations of BQ in the lower range (2–20 mg/l) had at least some inhibitory effect. In the concentration range of 2–15 mg/l BQ, the values for glucose consumption, Y_EtOH_ on consumed sugar, and BEY were at least 89 % of the corresponding values of the reference fermentation (Table 3). However, as indicated by Fig. 2b and by the low volumetric ethanol productivity values (Q = 0.17–0.35 g l^−1^ h^−1^) compared to that of the reference fermentation (Q = 0.75 g l^−1^ h^−1^) (Table 3), the time required to achieve complete glucose consumption and high ethanol yield was much longer when 2–15 mg/l BQ were present in the medium. For the highest concentration of BQ in Series B, 20 mg/l, the values for BEY and Q_EtOH_ were 4–7 % of that of the reference fermentation, while the glucose consumption reached 11 % of that observed for the reference fermentation. The discrepancy between ethanol formation and glucose consumption can be explained by a decrease in Y_EtOH_ on consumed glucose, as cultures with 20 mg/l BQ had a Y_EtOH_ on consumed glucose that was only 54 % of that of the reference fermentation.

With regard to the lowest concentration of DMBQ (20 mg/l), the glucose consumption rate (Fig. 2a) and the residual and consumed glucose (Table 3) were similar to that of the reference fermentation. However, with regard to ethanol formation, 20 mg/l DMBQ had a slight inhibitory effect, as shown by an 18 % decrease in Y_EtOH_ on consumed sugar, a 16 % decrease in BEY, and a 16 % decrease in Q_EtOH_ (Table 3). Obviously the presence of 20 mg/l DMBQ resulted in a shift from utilization of sugar for ethanolic fermentation to other metabolic activities. The higher concentrations of DMBQ were strongly inhibitory to *S. cerevisiae*, as shown by that the glucose consumption in cultures to which 0.2 or 1 g/l DMBQ was added was <20 % of that of the reference fermentation, and by that the BEY and Q_EtOH_ values were <10 % of that of the reference (Fig. 2a; Table 3). The Y_EtOH_ on consumed sugar was <50 % of that of the reference fermentation (Table 3), which explains why DMBQ had a larger negative impact on BEY and Q_EtOH_ than on the glucose consumption, which agrees with the results from the cultures with 20 mg/l DMBQ.

## Discussion

The sample matrix of lignocellulosic pretreatment liquids is complex as it contains aliphatic acids, uronic acids, aromatic acids, aldehydes, ketones, alcohols, and various saccharides (Du et al. 2010; Jönsson et al. 2013). Derivatization with DNPH increases the selectively for ketones and aldehydes (Brady and Elsmie 1926; Allen 1930), thus overcoming the complexity of the sample matrix. DNPH can be used to derivatize carbonyl compounds in aqueous samples (Kieber and Mopper 1990; Zwiener et al. 2002). Furthermore, it has previously been shown that DNPH reacts with benzoquinones (Fryling et al. 1995). In contrast, hydroquinones, which possess two hydroxyls as functional groups, cannot react with DNPH.

The signal intensities of derivatized DMBQ decreased during analysis. This could be the result of the instability of the hydrazone of DMBQ under acidic conditions due to increased electrophilicity at the first carbon of the benzene ring, which corresponds to a mechanism proposed by Kalia and Raines (2008). Considering the instability of the DMBQ hydrazone the data reported for DMBQ should be handled with care, and further investigations are necessary to obtain high-quality data for DMBQ.

Oxidation of lignin and lignin-derived compounds, such as *p*-hydroxybenzyl alcohols, *p*-hydroxybenzaldehydes and *p*-hydroxybenzoic acids, can result in the formation of *p*-benzoquinones (Sarkanen and Ludwig 1971; Saa et al. 1986). For example, Wozniak et al. (1989) investigated the use of chemical oxidants to generate *o*-quinones and *p*-quinones from lignin and lignin model compounds. Oxidation of various lignin samples and of syringyl-type compounds resulted in the formation of DMBQ (Wozniak et al. 1989). Pretreatment of lignocellulosic feedstocks under acidic conditions can result in formation of compounds with syringyl units (Martín et al. 2002a; Du et al. 2010; Mitchell et al. 2014) that could serve as precursors of DMBQ.

The concentrations of BQ and DMBQ found in the current study can be compared with reported concentrations of hydroquinone and 2,6-dimethoxyhydroquinone in quantifications based on GC–MS (gas chromatography–mass spectrometry) analysis of TMS (trimethylsilyl) derivatives. Larsson et al. (1999) reported 17 mg/l hydroquinone in an acid hydrolysate of Norway spruce. The highest concentration of hydroquinone found in the acid hydrolysates studied by Mitchell et al. (2014) was 6 mg/l, which was found in a hydrolysate prepared from bana grass. The hemicellulose hydrolysate of steam-treated birch contained 2 mg/l 2,6-dimethoxyhydroquinone (Buchert et al. 1990). Thus, reported concentrations of hydroquinones are roughly in the same range as the concentrations of benzoquinones reported in this work.

Larsson et al. (2000) studied twenty different aromatic compounds including BQ and showed that structurally similar compounds, such as BQ and hydroquinone, could have very different inhibitory effects on *S. cerevisiae*. Furthermore, Larsson et al. (2000) also showed that the polymerization of benzoquinone to high molecular weight polymers may occur in the presence of microbial growth medium and in the presence or absence of yeast, suggesting that metal ions may be responsible for catalyzing the polymerization reaction. Considering the complex matrix composition and the time periods for handling and storage of the pretreated lignocellulosic materials used in this study, it is not improbable that the concentrations of benzoquinones were far below the concentrations that were originally present directly after pretreatment of the lignocellulosic raw materials.

Cavka and Jönsson (2013) studied the effects of detoxification with sodium borohydride on selected inhibitors, and, assuming that benzoquinones could tentatively be present in lignocellulosic hydrolysates, found that treatment with sodium borohydride quantitatively reduced BQ to hydroquinone and reduced the concentration of DMBQ with 86 %. Detoxification with reducing agents, such as sodium borohydride (Cavka and Jönsson 2013) or the sulfur oxyanions dithionite and sulfite (Cavka et al. 2011), should be an efficient way to avoid problems with inhibitory benzoquinones.

The phenol-oxidizing enzyme laccase has successfully been used for detoxification of lignocellulosic hydrolysates prior to microbial fermentation. This is supported by observations that cover hydrolysates prepared from a wide variety of lignocellulosic feedstocks including willow (Jönsson et al. 1998), spruce wood (Larsson et al. 1999), sugarcane bagasse (Martín et al. 2002b; Chandel et al. 2007), and wheat straw (Jurado et al. 2009). Laccase treatment alone did little benefit to fermentation by *E. coli* of an inhibitory hydrolysate of sugarcane bagasse, but had a clear positive effect when used in combination with certain other treatment methods (Geddes et al. 2015). The positive effect of laccase treatment is associated with polymerization of small phenolic compounds to larger molecules and a decrease of the total content of phenolic compounds (Jönsson et al. 1998; Jurado et al. 2009). Although beneficial in many cases, comparisons of different detoxification methods indicate that laccase treatment does not remove all inhibitory compounds and that methods such as alkali treatment and ion exchange can give even better results (Larsson et al. 1999; Martín et al. 2002b; Chandel et al. 2007; Geddes et al. 1015). It is well known that laccase-catalyzed oxidation of certain phenolic compounds can result in formation of quinoid products (Lundquist and Kristersson 1985). BQ has been reported as a product of oxidation by laccase of 1,4-hydroquinone (Hahn et al. 2014). Therefore it is noteworthy in this context that while laccase typically has a general positive effect on the fermentability of lignocellulosic hydrolysates, which can be attributed to phenol polymerization, there is at least a theoretical risk with respect to hydroquinones that oxidation reactions to some extent can result in the formation of toxic benzoquinones.

The inhibitory effects of benzoquinones reported in this study are limited to studies of media where only one of the benzoquinones was present. In a lignocellulosic hydrolysate, many different substances would contribute to the total inhibitory effect, and there may also be synergetic effects between different inhibitory compounds (Zaldivar and Ingram 1999).

In conclusion, we developed an approach that was found to be useful for analysis of the presence of BQ and DMBQ in lignocellulosic hydrolysates. Using this approach, we could for the first time show the presence of benzoquinones in pretreated lignocellulose. BQ was found in all of the six lignocellulosic hydrolysates examined, and in at least four cases the concentrations were high enough (≥2 mg/l) to definitely contribute to the inhibition of yeast. Future efforts are needed to identify and quantify other benzoquinones than BQ and DMBQ in lignocellulosic hydrolysates, to understand the influence of the feedstock, the pretreatment conditions, and the stability of benzoquinones on the concentrations found in the hydrolysates, to study inhibitory effects of benzoquinones on other microorganisms than *S. cerevisiae*, and investigate how microorganisms can protect themselves against toxic benzoquinones.

## Abbreviations

ACN: acetonitrile
BEY: balanced ethanol yield
BQ: *p*-benzoquinone
DMBQ: 2,6-dimethoxybenzoquinone
DNPH: 2,4-dinitrophenylhydrazine
DW: dry weight
FIA: flow injection acquisition
GC-MS: gas chromatography–mass spectrometry
HMF: 5-hydroxymethylfurfural
HPLC: high-performance liquid chromatography
LOD: lower limit of detection
LOQ: limit of quantification
MRM: multiple reaction monitoring
MS: mass spectrometry
PID: proportional integral derivative
PL: pretreatment liquid
RID: refractive index detector
RT: room temperature
S/N: signal/noise
UHPLC-ESI-QqQ-MS: ultra-high performance liquid chromatography-electrospray ionization-triple quadrupole-mass spectrometry
UHPLC-Q-ToF-MS: ultra-high performance liquid chromatography-quadrupole-time-of-flight mass spectrometry
